# Time Course and Clinical Significance of Hematoma Expansion in Moderate-to-Severe Traumatic Brain Injury: An Observational Cohort Study

**DOI:** 10.1007/s12028-022-01609-w

**Published:** 2022-09-27

**Authors:** Alexander Fletcher-Sandersjöö, Charles Tatter, Jonathan Tjerkaski, Jiri Bartek, Marc Maegele, David W. Nelson, Mikael Svensson, Eric Peter Thelin, Bo-Michael Bellander

**Affiliations:** 1 Department of Neurosurgery, Karolinska University Hospital, Stockholm, Sweden; 2 Department of Clinical Neuroscience, Karolinska Institutet, Bioclinicum J5:20, 171 64 Solna, Stockholm, Sweden; 3 Department of Neurosurgery, Rigshospitalet, Copenhagen, Denmark; 4 Department for Trauma and Orthopedic Surgery, Cologne-Merheim Medical Center, Witten/Herdecke University, Cologne, Germany; 5 Institute for Research in Operative Medicine, Witten/Herdecke University, Cologne, Germany; 6 Department of Physiology and Pharmacology, Section of Perioperative Medicine and Intensive Care, Karolinska Institutet, Stockholm, Sweden; 7 Function Perioperative Care and Medicine, Karolinska University Hospital, Stockholm, Sweden; 8 Department of Neurology, Karolinska University Hospital, Stockholm, Sweden

**Keywords:** Hematoma expansion, Hemorrhage progression, Intracranial hemorrhage, Lesion progression, Progressive hemorrhagic injury, Traumatic brain injury

## Abstract

**Background:**

Preventing intracranial hematoma expansion has been advertised as a possible treatment opportunity in traumatic brain injury (TBI). However, the time course of hematoma expansion, and whether the expansion affects outcome, remains poorly understood. In light of this, the aim of this study was to use 3D volume rendering to determine how traumatic intracranial hematomas expand over time and evaluate its impact on outcome.

**Methods:**

Single-center, population-based, observational cohort study of adults with moderate-to-severe TBI. Hematoma expansion was defined as the change in hematoma volume from the baseline computed tomography scan until the lesion had stopped progressing. Volumes were calculated by using semiautomated volumetric segmentation. Functional outcome was measured by using the 12 month Glasgow outcome scale (GOS).

**Results:**

In total, 643 patients were included. The mean baseline hematoma volume was 4.2 ml, and the subsequent mean hematoma expansion was 3.8 ml. Overall, 33% of hematomas had stopped progressing within 3 h, and 94% of hematomas had stopped progressing within 24 h of injury. Contusions expanded significantly more, and for a longer period of time, than extra-axial hematomas. There was a significant dose–response relationship between hematoma expansion and 12 month GOS, even after adjusting for known outcome predictors, with every 1-ml increase in hematoma volume associated with a 6% increased risk of 1-point GOS deduction.

**Conclusions:**

Hematoma expansion is a driver of unfavorable outcome in TBI, with small changes in hematoma volume also impacting functional outcome. This study also proposes a wider window of opportunity to prevent lesion progression than what has previously been suggested.

**Supplementary Information:**

The online version contains supplementary material available at 10.1007/s12028-022-01609-w.

## Introduction

In traumatic brain injury (TBI), the primary injury can initiate events that lead to secondary brain damage [1]. Of the many potential secondary processes, hematoma expansion has been advertised as a possible therapeutic target, as it often occurs when patients are hospitalized, and there would be an excellent opportunity to intervene if proper treatment could be devised.

Although predictors of TBI-associated hematoma expansion are well studied [2], little is known about how these lesions progress over time and whether their progression affects outcome. One limitation that runs through the existing body of literature is the binary definition of hematoma expansion, based on absolute or proportional cut offs [3–13], with the threshold inducing a major source of discrepancy. In addition, the ABC/2 formula has often been used to calculate lesion size [3–6, 8, 10, 11, 13–17], even though it is inferior to 3D volume rendering for traumatic hematomas [18–20]. Contrast that with hemorrhagic stroke, in which the topography more resembles that of an ellipsoid and ABC/2 might be more suitable [21, 22]. Beyond these limitations, demonstrated associations between traumatic lesion progression and outcome have been restricted to univariate analyses [7, 23, 24], and it remains unclear whether hematoma expansion represents an inevitable stage in the natural history of traumatic bleeds or a secondary injury that can be prevented.

In light of the above, the aim of this study was to use 3D volume rendering to determine how traumatic intracranial hematomas expand over time and evaluate its impact on outcome.

## Methods

### Study Design and Setting

This was a single-center, population-based, observational cohort study. Adults (≥ 15 years) with moderate-to-severe TBI who were admitted to the Karolinska University Hospital between 2006 and 2019 were eligible for inclusion. Moderate-to-severe TBI was defined as Glasgow Coma Scale (GCS) [25] score of 3–13, with the last known GCS prior to intubation used if a patient was intubated on trauma center arrival. The study hospital is the only level I trauma center equivalent in the region and offers neurosurgical and neurointensive care to 2.4 million people. Patients were excluded if no hemorrhagic lesion was detected, if their time of injury was unknown, if they had suffered a penetrating brain injury, or if their first or second computed tomography (CT) scan was performed more than 12 or 48 h after injury, respectively. The study was approved by the Swedish Ethical Review Authority (Dnr: 2019–04476), who waived the need for informed consent, and was performed in accordance with the ethical standards as laid down in the 1964 Declaration of Helsinki and its later amendments.

### Data Collection

Patients were identified from a local trauma database that includes all patients admitted to the hospital with TBI. Clinical data were reviewed by using the medical records software TakeCare (CompuGroup Medical Sweden AB, Farsta, Sweden), and imaging data were retrieved from the radiological management software Sectra Picture Archiving and Communication System IDS7 (Sectra AB, Linköping, Sweden). Collected data included demographics, comorbidities, injury time and mechanism, clinical status on admission, radiographic data from all CT scans performed during hospitalization, treatment, and 12 month Glasgow Outcome Scale (GOS) [26]. Patients with an extracranial Abbreviated Injury Scale [27] ≤ 2 were classified as isolated TBI. The main outcome was hematoma expansion, defined as the increase in hematoma volume (in ml) from the baseline CT scan until the lesion had stopped progressing. This was calculated for contusions, subdural hematomas (SDHs), epidural hematomas (EDHs), and for all lesions combined.

### Hematoma Volume Calculations

On arrival to the trauma center, each patient generally underwent a baseline CT scan and follow-up imaging at least 6 and 24 h later, respectively. Hematoma volumes were calculated from CT scans by using a semiautomated volumetric segmentation tool developed by Sectra AB and built into the radiological management system Picture Archiving and Communication System IDS7 version 21.1.8. Using this tool, the lesions were manually identified, and their volumes were automatically calculated on the basis of adjacent voxels of similar Hounsfield units [28]. The lesion maps were then reviewed and manually corrected, if needed, before final 3D rendered hematoma volumes were extracted. We included all CT scans performed until each patient’s hematomas had stopped expanding. The extracted volumes were rounded to the nearest 0.1 ml, with hematoma expansion defined as any increase in hematoma size [16, 29]. In the case of multiple lesions of the same type, the volumes were summed. Volume calculations were performed by three of the authors (AFS, CT, JT), with excellent interobserver variability (see “Results” section). A hematoma was determined to have stopped expanding when two consecutive CT scans showed the same volume for the lesion in question. To reduce the influence of surgical treatment, only nonoperated lesions were assessed. For traumatic subarachnoid hemorrhage (tSAH), volume calculation was not possible, and expansion was instead dichotomized as determined by a specialist in neuroradiology. Intraventricular hemorrhage (IVH) expansion was not evaluated because of repeated uncertainty of whether an increase was due to tSAH redistribution.

### Statistics

As all continuous data significantly deviated from a normal distribution pattern (Shapiro-Wilks test *p* value < 0.05), these data are presented as median (range), and categorical data are presented as numbers (proportion). Hematoma volumes are also presented as mean (standard deviation) to illustrate significant changes.

To determine the reliability and reproducibility of the hematoma volume calculations, interobserver agreement between the three assessors was estimated by using intraclass correlation coefficients (ICCs), and their 95% confident intervals were calculated for 30 randomly selected patients, based on a mean-rating (*k* = 3), absolute-agreement, two-way, mixed-effects model. Generally, ICC ranges from 0 to 1, with interobserver agreement classified into poor (< 0.50), moderate (0.5–0.75), good (0.75–0.90), and excellent (> 0.90) [30].

The Kruskal–Wallis test was used to compare hematoma expansion between hemorrhage subtypes. Univariable and multivariable proportional odds logistic regressions were used to assess the impact of hematoma expansion on 12 month GOS. In the multivariable model, we included variables from the CT and core International Mission for Prognosis and Analysis of Clinical Trials in TBI (IMPACT) model previously shown to be major predictors for TBI outcome (age, GCS, pupillary status, Marshall CT classification, presence of tSAH and EDH, as well as oxygen saturation and blood pressure at the scene of accident [31]). In the step-down multivariable model, variables significant in the univariable analysis were sequentially omitted, based on the highest *p* value, until all values in the model were significant. Nagelkerke’s pseudo *R*^2^ was used to illustrate the explained variance in the univariable model. In the multivariable model, listwise deletion was used because only 0.3% of data were missing.

All analyses were conducted by using the statistical software program R (version 4.0.3). Statistical significance was set at *p* < 0.05.

## Results

### Baseline Data

**Fig. 1:**
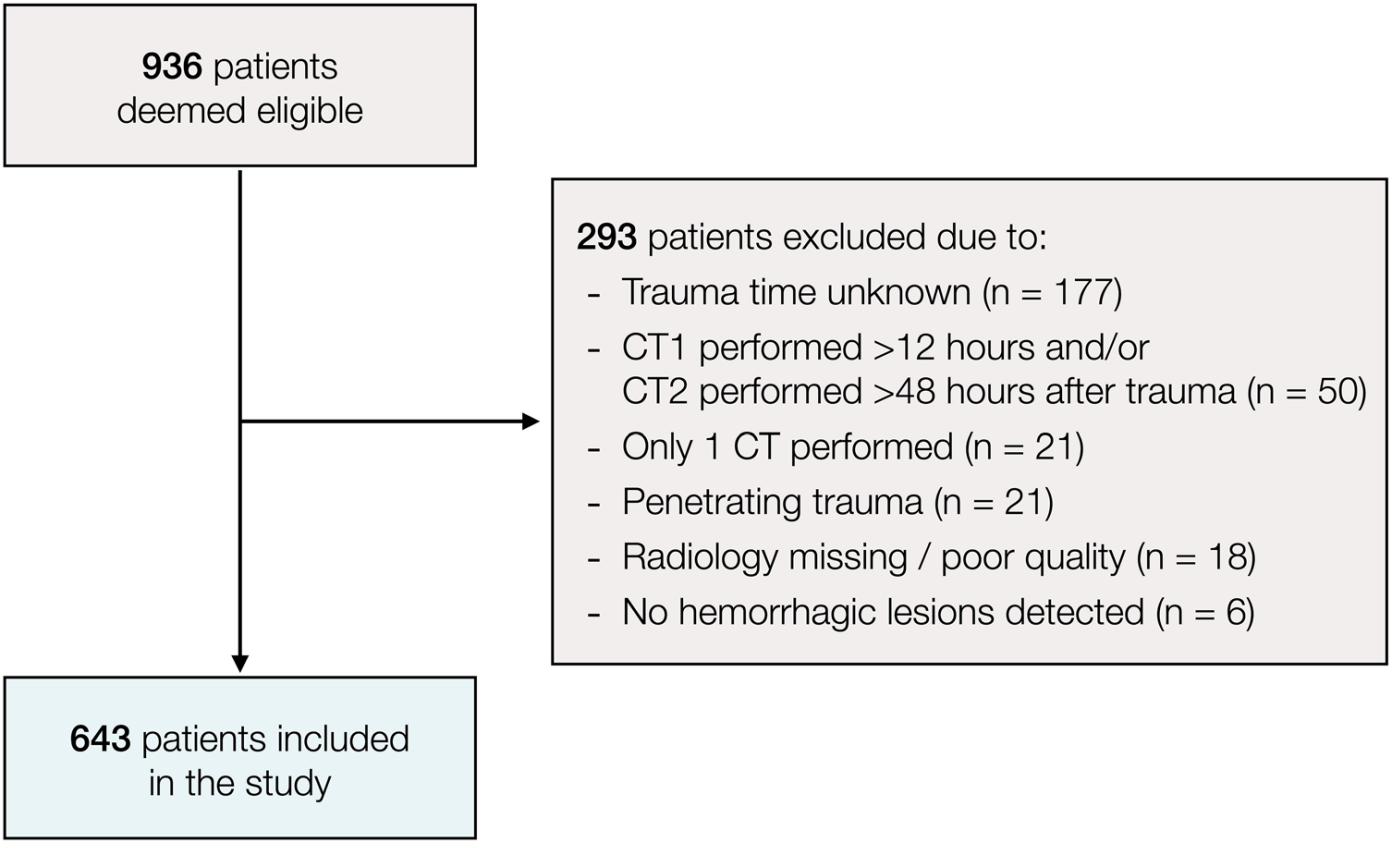
Flowchart illustrating the patient selection process. CT, computed tomography

Of 936 eligible patients, 643 were included in the study (Fig. 1). The median GCS on arrival was 7 (interquartile range 3–11), and same-level falls were the major cause of injury (*n* = 260, 40%). Contusions were the most common lesion type (*n* = 491, 76%) followed by tSAH (*n* = 483, 75%). Ninety percent of patients were intubated, invasive neuromonitoring was used in 65%, and hematoma evacuation was performed in 44%. The median 12-month GOS was 4, which corresponds to “moderate disability” (Table 1).

**Table 1:** Baseline, treatment, and outcome data

Variable	Patients (*n* = 643)
Baseline	
Age (years), median (IQR) (years)	47 (28 to − 62)
Male sex, *n* (%)	478 (74%)
Pre-injury anticoagulation, *n* (%)	20 (3.2%), (10 missing)
Pre-injury antiplatelet drug, *n* (%)	44 (7.0%), (10 missing)
Isolated TBI, *n* (%)	455 (71%)
Injury mechanism, n (%)	
Fall from standing position, n (%)	260 (40%)
Fall from height, *n* (%)	80 (12%)
Traffic accident, *n* (%)	206 (32%)
Struck by blunt object, *n* (%)	82 (13%)
Unknown, *n* (%)	15 (2.3%)
GCS on admission, median (IQR)	7.0 (3.0 to − 11)
Moderate TBI, *n* (%)	207 (32%)
Severe TBI, *n* (%)	436 (68%)
Unilateral/bilateral pupil unresponsiveness, *n* (%)	77 (12%)/66 (10%)
Bilateral pupil unresponsiveness, *n* (%)	66 (10)
Laboratory values on admission	
Prothrombin time (INR), median (IQR)	1.1 (1.0–1.3), (211 missing)
APTT (seconds), median (IQR) (seconds)	32 (28–36), (212 missing)
Platelet count (× 10^9^/L), median (IQR)	192 (153–231), (220 missing)
Treatment, n (%)	
Tranexamic acid within 3 h of injury, n (%)	46 (7.2%)
Intubation, n (%)	580 (90%)
Invasive neuromonitoring, n (%)	421 (65%)
Hematoma evacuation, *n* (%)	285 (44%)
Contusion evacuation, *n* (%)	51 (7.9%)
EDH evacuation, *n* (%)	95 (15%)
SDH evacuation, *n* (%)	185 (29%)
Outcome	
30 day mortality, *n* (%)	74 (12%)
12 month mortality, *n* (%)	114 (18%)
12 month GOS, median (IQR)	4.0 (3.0 to − 4.0)

*APTT* activated partial thromboplastin time, *EDH* epidural hemorrhage, *GCS* glasgow coma scale, *GOS* glasgow outcome scale, *INR* international normalized ratio, *IQR* interquartile range, *n* number, *TBI* traumatic brain injury, *SDH* subdural hemorrhage

### Interobserver Volume Calculation Agreement

There was excellent interobserver agreement for hematoma volume calculations, with an ICC of 0.98 (95% confidence interval 0.97–0.99).

### Hematoma Expansion Over Time

**Fig. 2:**
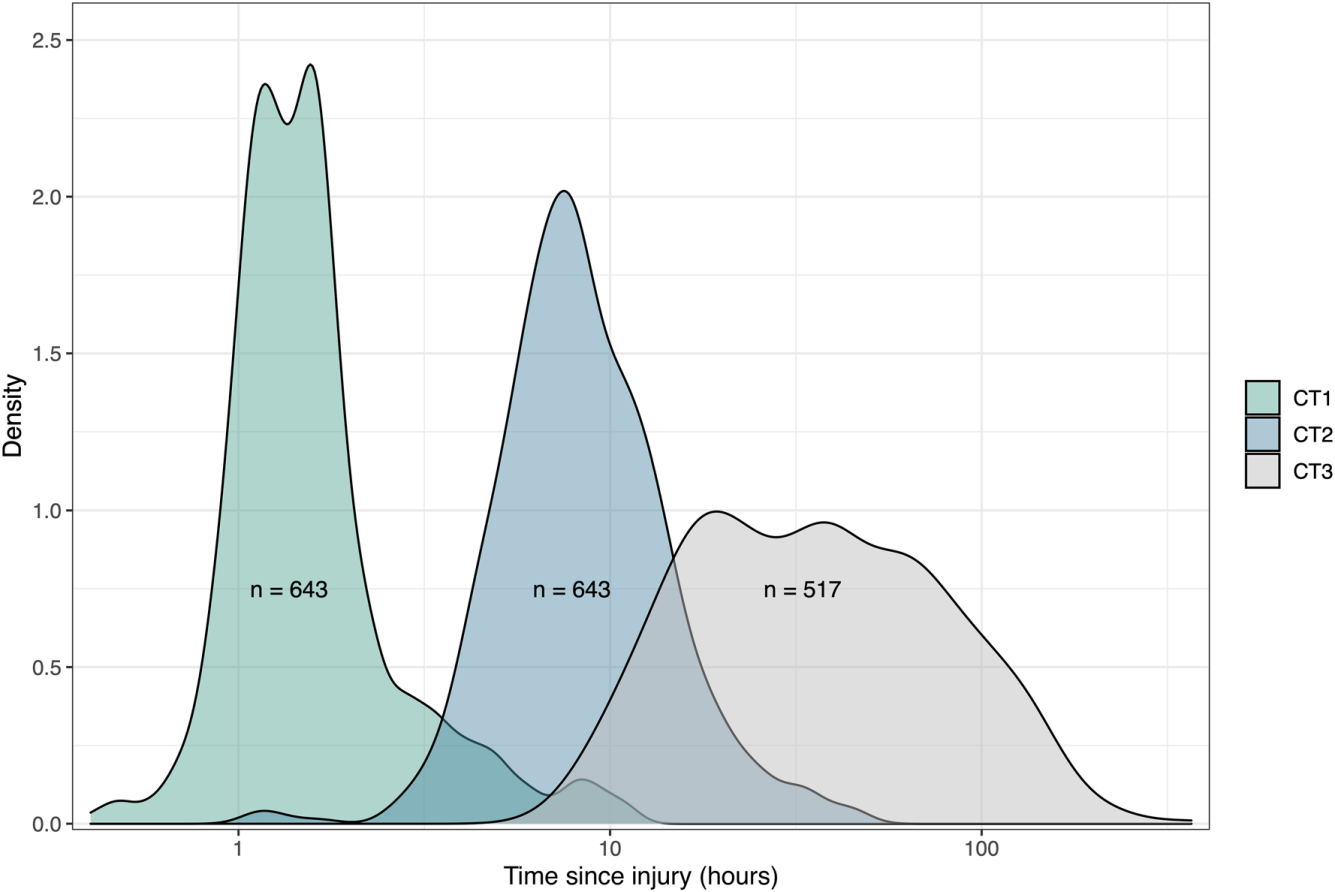
Density plot illustrating when the first, second, and third computed tomography (CT) scans were performed in relation to the time of injury. The *x*-axis has a logarithmic scale due to its nonparametric distribution

**Fig. 3:**
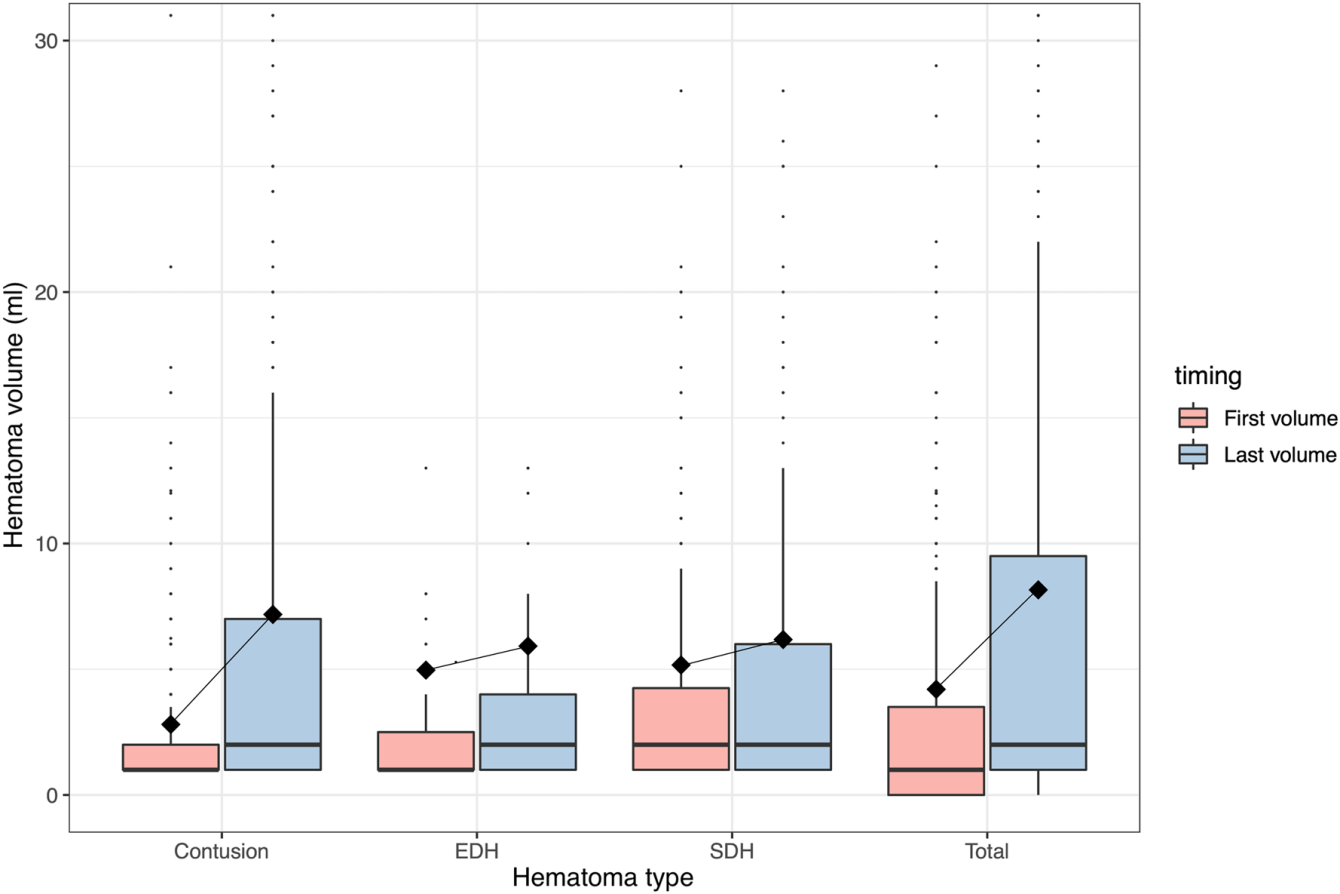
Box plot showing the first and final lesion volumes for contusions, epidural hematomas (EDH), subdural hematomas (SDH), and all lesions combined. The diamond shows the mean, the center line in the box shows the median, and the bottom and top of the box show the 1st and 3rd quantiles for the data. The lines that extend from the box represent the expected variation of the data, and the points extending beyond these lines are outliers

**Fig. 4:**
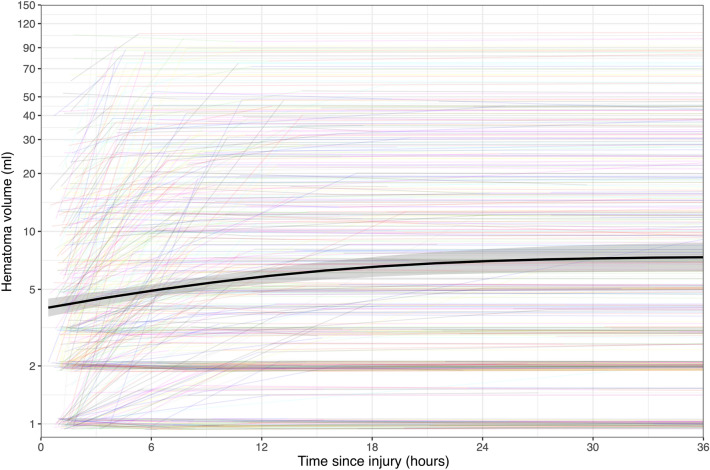
Spaghetti plot showing total lesion volume over time for each individual patient, with each colored line representing a patient’s lesion volume as determined by the latest available computed tomography (CT) scan. To reduce the influence of CT section thickness, small lesions (< 1 ml) were classified as “1 ml” even if they decreased in size during the first 24 h. The black line is a Locally Weighted Scatterplot Smoothing (LOWESS) curve, and the shaded area surrounding it indicates 95% confidence intervals. The *y*-axis has a logarithmic scale

The median time from injury to the first CT scan was 1.4 h (Fig. 2), and the mean baseline hematoma volume was 4.2 ± 11 ml. The mean hematoma expansion, defined as the increase in hematoma volume from the baseline CT scan until the lesion had stopped progressing, was 3.8 ± 9.2 ml (Table 2), with 394 patients (61%) showing some form of hematoma expansion after their first CT scan. Contusions expanded significantly more than SDH and EDH (*p* < 0.001) (Fig. 3). As illustrated in Fig. 4, hematoma expansion slowed exponentially over time, with close to no volume increase occurring 24 h after trauma.

**Table 2:** Radiographic data

Variable	Patients (*n* = 643)
Hours from injury to first CT scan, median (IQR)	1.4 (1.1 to − 1.9)
Hours from injury to second CT scan, median (IQR)	8.0 (6.1 to − 12)
Hours from injury to third CT scan, median (IQR)	35 (19 to − 65)
Marshall CT classification on admission, median (IQR)	3.0 (2.0 to − 5.0)
Lesion types	
Contusion, *n* (%)	491 (76%)
Supratentorial, n (%)	482 (75%)
Infratentorial, n (%)	41 (6.4%)
SDH, *n* (%)	458 (71%)
EDH, *n* (%)	157 (24%)
tSAH, *n* (%)	483 (75%)
IVH, *n* (%)	194 (30%)
Baseline hematoma volume (mL), mean ± SD (ml)	4.2 ± 11
Contusion volume (mL), mean ± SD (ml)	2.8 ± 6.9
SDH volume (mL), mean ± SD (ml)	5.2 ± 11
EDH volume (mL), mean ± SD (ml)	5.0 ± 16
Hematoma expansion (mL), mean ± SD (ml)	3.8 ± 9.2
Contusion expansion (mL), mean ± SD (ml)	4.7 ± 10
SDH expansion (mL), mean ± SD (ml)	1.3 ± 4.3
EDH expansion (mL), mean ± SD (ml)	1.5 ± 5.2

*CT* computed tomography, *EDH* epidural hemorrhage, *GOS* glasgow outcome scale, *IQR* interquartile range, *IVH* intraventricular hemorrhage, *mL* milliliters, *n* number, *tSAH* traumatic subarachnoid hemorrhage, *SD* standard deviation, *SDH* subdural hemorrhage, *tSAH* traumatic subarachnoid hemorrhage

**Fig. 5:**
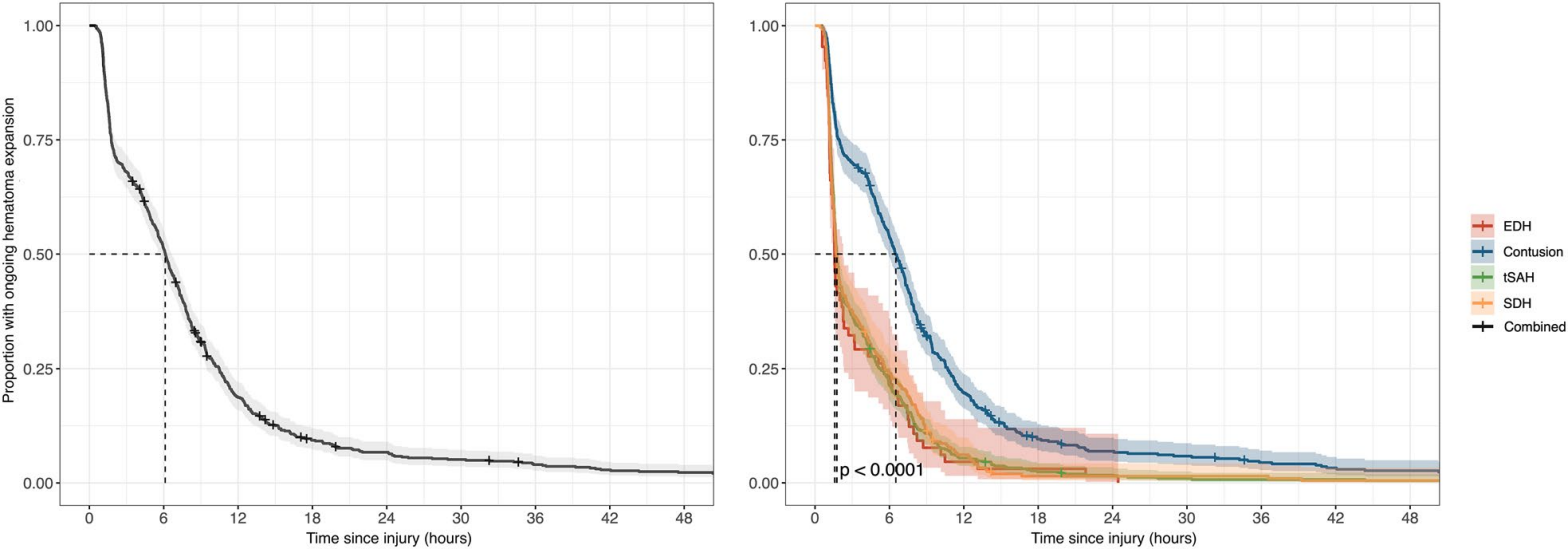
Kaplan Meier curve showing lesion progression time for contusions, epidural hematomas (EDH), subdural hematomas (SDH), and traumatic subarachnoid hematomas (tSAH) (right) and for all lesions combined (left). Lesion progression was defined as any expansion of existing hemorrhagic lesions or the appearance of a new lesion, and a lesion was determined to have stopped progressing when two consecutive computed tomography scans showed the same volume for the lesion in question. The shaded areas indicate 95% confidence intervals. The dotted vertical lines show the median lesion progression times for contusions (6.27 h), SDH (1.68 h), EDH (1.58 h), tSAH (1.76 h), and all lesions combined (6.0 h)

Figure 5 illustrates when hematomas verifiably had stopped expanding, i.e., when two consecutive CT scans showed the same volume. Overall, 33% of lesion had stopped expanding within 3 h, 66% within 8 h, 94% within 24 h, and 98% within 48 h of injury. The median time to halted expansion for all hematomas combined was 6.0 h (interquartile range 1.8–10), with contusions progressing for a longer time than extra-axial hematomas (*p* < 0.001).

### Clinical Significance of Hematoma Expansion

Hematoma expansion was significantly associated with 12 month GOS in both the univariable analysis (Table 3) and after adjusting for other known outcome predictors in the multivariable model (Table 4). The odds ratio for hematoma expansion was 0.94, indicating that for each 1-ml increase in hematoma size, patients were 6% more likely have a 1-point decrease in GOS score. Of note, the association between hematoma expansion and outcome seemed to be driven primarily by the expansion of contusions and SDH rather than EDH (Supplementary Table 1). The results from the univariable analyses also remained unchanged even when all patients who underwent some form of hematoma evacuation were excluded (Supplementary Table 2).

**Table 3:** Univariable proportional odds logistics regression predicting 12-month GOS

Variable	*p*-value*	Nagelkerke’s pseudo*-R*^2^	OR (95% CI)
IMPACT model			
Age (years)	** < 0.001**	0.095	0.97 (0.96 to − 0.98)
GCS on admission	** < 0.001**	0.127	1.20 (1.16 to − 1.25)
Unilateral pupil unresponsiveness	**0.001**	0.017	0.51 (0.33 to − 0.77)
Bilateral pupil unresponsiveness	** < 0.001**	0.093	0.16 (0.10 to − 0.26)
Marshall CT classification	**0.006**	0.013	0.87 (0.79 to − 0.96)
Subarachnoid hemorrhage	**0.016**	0.010	0.68 (0.50 to − 0.93)
Epidural hemorrhage	** < 0.001**	0.062	2.85 (2.04 to − 3.98)
﻿ Oxygen saturation at SoA (%)	** < 0.001**	0.032	1.05 (1.02 to − 1.07)
Blood pressure at SoA (mm Hg)	** < 0.001**	0.058	0.99 (0.98 to − 0.99)
New variable			
Hematoma expansion (mlL)	** < 0.001**	0.078	0.94 (0.93 to − 0.96)

OR < 1 means that the presence of, or increase in, the explanatory variable leads to decreased GOS (i.e., a more unfavorable outcome)

*CI* confidence interval, *CT* computed tomography, *GCS* glasgow coma scale, *GOS* glasgow outcome scale, *IMPACT* International Mission for Prognosis and Analysis of Clinical Trials in TBI, *mL* milliliters; *mmHg* millimeters of mercury, *OR* odds ratio, *SoA* scene of accident

*Bold text in the *p* -value column indicates a statistically significant correlation (*p* < 0.05). OR < 1 means that the presence of, or increase in, the explanatory variable leads to decreased GOS (i.e. a more unfavorable outcome)

**Table 4:** Multivariable proportional odds logistics regression predicting 12-month GOS: final results for step-down model

Variable	*p*-value*
Age	< 0.001
GCS on admission	< 0.001
Hematoma expansion	< 0.001
Bilateral/unilateral pupil unresponsiveness	< 0.001/0.026
Unilateral pupil unresponsiveness	0.026
Epidural hemorrhage	< 0.001

Nagelkerke’s pseudo*-R*^2^ = 0.347

*CT* computed tomography, *GCS* glasgow coma scale, *GOS* glasgow outcome scale, *mL* milliliters, *LPT* lesion progression time

^*^Bold text in the *p*-value column indicates a statistically significant correlation (*p* < 0.05)

**Fig. 6:**
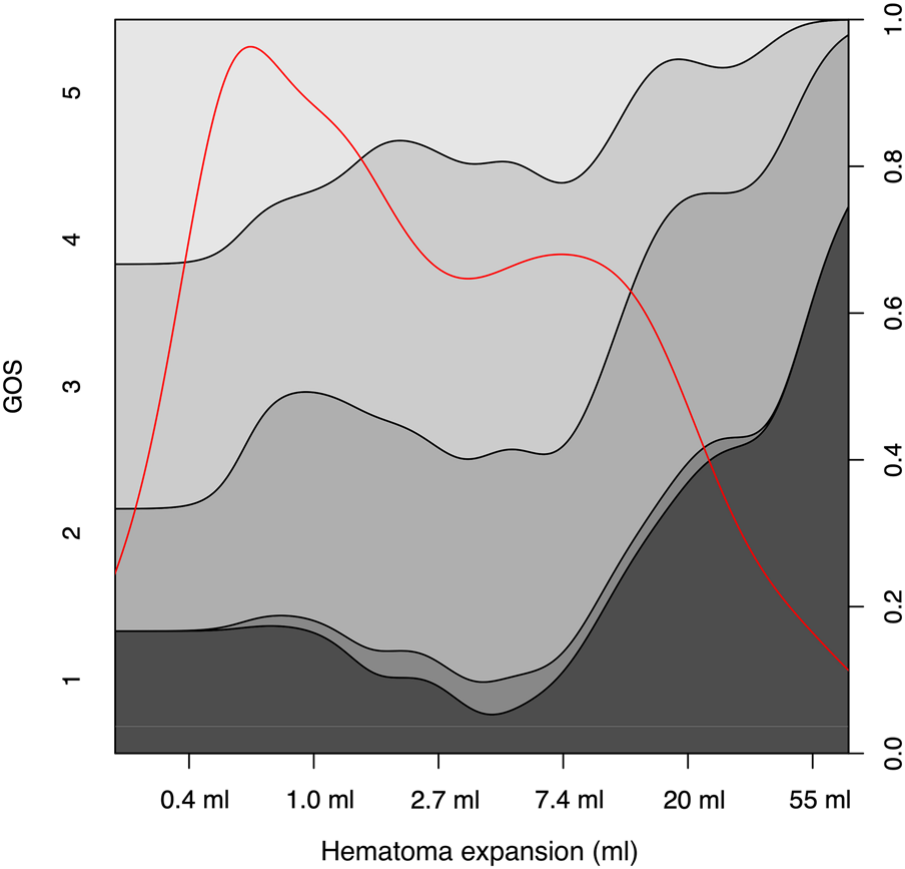
Conditional density plot showing the association between the different stages of Glasgow Outcome Score (*y*-axis) and hematoma expansion. The *x*-axis has a logarithmic scale because of its nonparametric data distribution. The red line illustrates the data distribution

Figure 6 is a conditional density plot showing the relationship between hematoma expansion and 12-month GOS, with larger hematoma expansion (*x*-axis) correlating with a higher degree of patients with lower GOS score (*y*-axis). Interestingly, even minor hematoma expansion (0.4 ml to 2.7 ml) affected the distribution of patients who had a GOS score of 4–5, indicating that small changes in hematoma volume also impact functional outcome, albeit not mortality.

## Discussion

This study of 643 patients with moderate-to-severe TBI demonstrated an independent dose–response relationship between hematoma expansion and functional outcome, with every 1 ml increase in volume conferring a 6% increased risk of 1-point GOS deduction. This clinical significance was evident even for minor changes in hematoma volume, highlighting its importance as a therapeutic opportunity in TBI management. Moreover, a time window of hematoma progression was identified.

This is the first study to identify a dose–response response relationship between the magnitude of hematoma expansion and functional outcome. The results are supported by previous studies that have used dichotomized definitions of hemorrhage progression. Juratli et al. [23] showed that patients with contusion progression were more likely to have an unfavorable modified Rankin Scale at follow-upClick or tap here to enter text., Cepeda et al. [7] identified an association between lesion progression and 6-month GOSClick or tap here to enter text., and Qureshi et al. [24] reported a higher proportion of patients with unfavorable 6-month extended GOS in those with lesion progressionClick or tap here to enter text.. Although these studies demonstrated univariate associations between lesion progression and functional outcome, they did not remain significant in multivariate models, leading to a belief that lesion progression maybe only represented TBI severity rather than had an impact on outcome [2]. It is therefore interesting that hematoma expansion was independently associated with 12 month GOS in our study, as this is consistent with a hypothesis that lesion expansion is a driver, and not simply a marker, of poor outcome. Hematoma expansion is, so far, the only independent TBI outcome predictor that is amenable to treatment and thus a potential therapeutic target. In addition, even small increases in hematoma expansion affected long-term GOS, further highlighting its importance as a therapeutic opportunity in TBI management. Because of its correlation with outcome, and the potential to intervene to prevent its occurrence, hematoma expansion might also be a suitable surrogate end point for trials of hemostatic agents in TBI that are underpowered for functional outcome.

This study also confirms previous observations that hematoma expansion is more common in contusions than extra-axial hematomas [2, 14, 15, 29]. Our mean contusion volume increase of 4.7 ml is comparable to the 6.0 ml seen in the control group of a recent Collaborative European NeuroTrauma Effectiveness Research in TBI (CENTER-TBI) study on the effects of antiplatelet therapy on contusion expansion [29]. The differences in lesion progression time and volume between contusions and extra-axial hematomas may be due to their underlying pathophysiology; although the expansion of extra-axial hematomas can be credited to bleeding from damaged vessels, contusion progression has also been attributed to the effects of a traumatic penumbra surrounding the lesion, in which molecular processes may lead to delayed microvessel structural failure and bleeding progression, even in regions that appear to be unaffected on the first CT scan [32].

We also found that 33% of hematomas had stopped progressing within 3 h, 66% within 8 h, 94% within 24 h, and 98% within 48 h of injury. This, together with our identification of a trend toward decreasing lesion expansion over time (Fig. 5), sheds light on a potential time window for interventions that target hematoma expansion. This is supported by results from the CRASH-3 trial of tranexamic acid in TBI [33], which found that early treatment conferred the greatest outcome benefit. However, although the CRASH-3 study employed a time window for eligibility of 3 h, our finding that many lesions progress beyond this limit might enable future studies aimed at reducing hemorrhage progression to expand this therapeutic window, especially in contusion subgroups.

### Limitations

Requiring at least 2 CT scans within 48 h tended to exclude the most severely injured patients, who passed away before a second CT scan could be performed, as well as the less injured patients who did not need a second CT scan within this time frame. Highlighting this, 17 out of the 21 patients excluded due to only undergoing one CT scan performed passed away during hospitalization. Secondly, only nonoperated lesions were assessed for hematoma expansion, which tended to exclude large SDHs and EDHs because they are more likely to be surgically treated. This likely contributed to the fact that contusions appeared to progress for a longer period of time and to a greater extent than extra-axial hematomas. Thirdly, although most studies have used a dichotomized definition of hematoma expansion [3–11], we chose to define lesion progression as *any* increase in hematoma size (with volumes rounded to the nearest 0.1 ml). This has been done previously, for example, in a recent CENTER-TBI study [16, 29], and is, in our opinion, appropriate when volumetric segmentation is used instead of the ABC/2 formula because it allows for detection of more discrete volume changes [18] and isn’t affected by hematoma redistribution. However, this likely means that there were instances when stable hematomas were falsely classified as having shown small increases in size. To account for this, we used hematoma expansion as a continuous variable in the regression analysis, making it independent of how we defined lesion progression. Lesion progression time was also dependent on the timing of the CT scans, and we therefore likely overestimated the duration of lesion progression. For example, the fact that the median lesion progression time was 6.0 h might reflect our tendency to obtain a 6 h follow-up scan as opposed to a physiologic process dictating the natural progression of hematomas. It was also not possible to quantatively determine SAH progression, and the specialist in neuroradiology may have made some misclassifications due to hematoma redistribution. Lastly, we refrained from identifying predictors of hematoma expansion, as we considered it to be beyond the scope of this article, but plan on doing so in a future study. Despite these limitations, our study draws strength from the large study population, volumetric calculation of lesion sizes, excellent interobserver hematoma volume agreement, and continuous definition of hematoma expansion.

## Conclusions

The aim of this study was to use 3D volume rendering to assess the time course and clinical significance of intracranial lesion progression in moderate-to-severe TBI. A significant dose–response relationship between hematoma expansion and 12-month GOS was identified, highlighting its importance as a therapeutic target in TBI management. In addition, this study showed that even small changes in hematoma volume carry clinical significance and identified a wider window of opportunity to prevent lesion progression than what has been previously suggested.

## Supplementary Information

Below is the link to the electronic supplementary material.

Supplementary file1 (DOCX 15 kb)
