# Supplementary material for: Time Course and Clinical Significance of Hematoma Expansion in Moderate-to-Severe Traumatic Brain Injury: An Observational Cohort Study
Source: Neurocrit Care. 2022 Sep 27;38(1):60–70. doi: 10.1007/s12028-022-01609-w (PMC9935722; doi:10.1007/s12028-022-01609-w)
Supplement: Supplementary file 1 — Supplementary file1 (DOCX 15 kb) [file 12028_2022_1609_MOESM1_ESM.docx]

**Supplementary table 1: Univariable proportional odds logistics regression predicting 12-month GOS for hematoma subgroups**

| **Variable** | **p-value** | **Nagelkerke’s pseudo-R^2^** | **OR (95% CI)** |
| --- | --- | --- | --- |
| Contusion expansion (mL) | **< 0.001** | 0.096 | 0.97 (0.96 – 0.98) |
| SDH expansion (mL) | **0.006** | 0.037 | 0.91 (0.85 – 0.97) |
| EDH expansion (mL) | **0.290** | 0.020 | 1.04 (0.97 – 1.11) |

Abbreviations: CI = confidence interval; EDH = epidural hematoma; mL = milliliters; OR = odds ratio; SDH = subdural hematoma. Bold text in the p-value column indicates a statistically significant correlation (p < 0.05). OR < 1 means that the presence of, or increase in, the explanatory variable leads to decreased GOS (i.e. a more unfavorable outcome).

**Supplementary table 2: Univariable proportional odds logistics regression predicting 12-month GOS in non-operated patients**

| **Variable** | **p-value** | **Nagelkerke’s pseudo-R^2^** | **OR (95% CI)** |
| --- | --- | --- | --- |
| **IMPACT model** |  |  |  |
| Age (years) | **< 0.001** | 0.101 | 0.97 (0.96 – 0.98) |
| GCS on admission | **< 0.001** | 0.091 | 1.16 (1.10 – 1.23) |
| Unilateral pupil unresponsiveness | **< 0.001** | 0.049 | 0.26 (0.14 – 0.50) |
| Bilateral pupil unresponsiveness | **< 0.001** | 0.047 | 0.19 (0.08 – 0.43) |
| Marshall CT classification | **< 0.001** | 0.041 | 0.68 (0.56 – 0.83) |
| Subarachnoid hemorrhage | **< 0.001** | 0.036 | 0.43 (0.27 – 0.69) |
| Epidural hemorrhage | **< 0.001** | 0.034 | 2.41 (1.45 – 4.00) |
| ﻿Oxygen saturation at SoA (%) | **0.029** | 0.018 | 1.04 (1.00 – 1.07) |
| Blood pressure at SoA (mmHg) | **< 0.001** | 0.065 | 0.98 (0.98 – 0.99) |
| **New variable** |  |  |  |
| Hematoma expansion (mL) | **< 0.001** | 0.009 | 0.93 (0.91 – 0.96) |

Abbreviations: CI = confidence interval; CT = computed tomography; GCS = Glasgow Coma Scale; GOS = Glasgow Outcome Scale; mL = milliliters; mmHg = millimeters of mercury; OR = odds ratio; SoA = scene of accident. Bold text in the p-value column indicates a statistically significant correlation (p < 0.05). OR < 1 means that the presence of, or increase in, the explanatory variable leads to decreased GOS (i.e. a more unfavorable outcome).
